# Anxiety and initial value dependence in startle habituation

**DOI:** 10.1111/psyp.14071

**Published:** 2022-04-12

**Authors:** Jules Alex Faunce, Terry D. Blumenthal, Christian E. Waugh

**Affiliations:** ^1^ Department of Psychology Virginia Polytechnic Institute and State University Blacksburg Virginia USA; ^2^ Department of Psychology Wake Forest University Winston‐Salem North Carolina USA

**Keywords:** anxiety, habituation, initial value dependence, reactivity, startle

## Abstract

Studies suggest that deficits in startle reflex habituation occur in trait and clinical anxiety. Measures of habituation are affected by the magnitude of the initial response, with larger initial responses predicting a steeper decline in response over repeated trials. This relationship between initial value and change, commonly called the Law of Initial Value or initial value dependence (IVD), has been partialled out as a covariate in habituation research, but variation in IVD may be informative in itself, reflecting differences in physiological reactivity. The present study explored how trait anxiety and contextual anxiety relate to habituation kinetics of the startle eyeblink response: initial value, linear habituation slope, and the relationship between them (IVD). Participants (n = 31; 15 Control, 16 Contextual Anxiety [CA]) were exposed to two blocks of acoustic startle stimuli, and CA participants were warned that they may receive an electrical shock to the wrist during block 2. Trait anxiety did not predict habituation slope, but it did predict a weaker IVD relationship, meaning that high initial startle magnitude was less predictive of a steep response decline in trait‐anxious subjects. Meanwhile, CA did not impact startle habituation or IVD. The results suggest that individual differences in trait anxiety are related to the relationship between initial physiological response magnitude and subsequent change in response. IVD in startle habituation may thus serve as a better biomarker of healthy emotional responding than startle habituation per se.

## Abstract 2

Startle reflex habituation relates to emotion regulation, but this relationship may be more meaningful when habituation is considered in the context of initial reactivity, referred to as initial value dependence (IVD). IVD is related to trait anxiety, and may also predict the efficacy of emotion regulation more generally.

## 1 INTRODUCTION

Anxiety is related to physiological responses to stimuli, including initial response magnitude (McTeague et al., 2011; McTeague & Lang, 2012), and response change (e.g., habituation; Campbell et al., 2014). Response change is often inversely related to initial response magnitude, a phenomenon known as initial value dependence (abbreviated IVD; Jin, 1992). Statistical treatment of IVD varies, with many researchers partialling the effect out of their analyses (Jin, 1992). However, IVD effects may reflect physiological reactivity (Duffy, 1962; Jamieson, 1993; Scher et al., 1985), which in turn relates to emotion regulation (Waugh et al., 2008). The present study assessed the relationship between IVD in startle habituation and trait and contextual anxiety.

### 1.1 Anxiety and startle responding

Anxiety is a feeling of unease associated with uncertain outcomes. It is valuable for survival and functioning, but resource‐intensive. Therefore, a person's anxious response to a stressor should adapt appropriately to emotional context. Chronic problems with physiological flexibility predict poorer health outcomes (Pine et al., 2001; Waugh et al., 2008), so flexibility in physiological responding has important applications in clinical psychology.

A growing body of research supports the importance of peripheral physiology in the study of anxiety (Benke et al., 2015; Seligowski et al., 2016; Yang & Friedman, 2017). Of particular interest to the present study is the startle reflex, an involuntary motor reaction that interrupts ongoing processes and redirects attention to sudden and potentially dangerous stimuli (Blumenthal, 2015). The preferred startle response measure in humans is the reflexive eyeblink, measured via electromyographic (EMG) activity from the orbicularis oculi muscle shortly after startle stimulus onset (in most cases, a short burst of sound; Blumenthal, 2015; Blumenthal et al., 2005).

In the primary acoustic startle eyeblink pathway, information travels via auditory afferent fibers to the nucleus reticularis pontis caudalis (nRPC), and then to the facial motor nucleus, resulting in orbicularis oculi muscle contraction (Davis, 2006). The low number of synapses in this reflex arc makes startle eyeblink EMG a relatively direct measure of brainstem activity (Davis, 2006). Of additional interest are brain areas projecting to this pathway: the amygdala, hippocampus, bed nucleus of the stria terminalis (BNST; Lee & Davis, 1997), and the anterior cingulate cortex (Medford & Critchley, 2010). All of these regions are involved in anxious responding (Davis & Whalen, 2001; Lee & Davis, 1997; Walker et al., 2003). Accordingly, the startle reflex varies with state, trait, and clinical measures of anxiety (Gorka et al., 2013; Grillon, 2002; Grillon et al., 2008; Grillon & Ameli, 1998; Michopoulos et al., 2015).

#### 1.1.1 Startle habituation

Startle magnitude decreases with stimulus repetition, through a nonassociative learning process called habituation (Blumenthal, 1997; Lane et al., 2013; Rankin, 2009; Rankin et al., 2009; Thompson & Spencer, 1966). This process results from synaptic depression in sensory neurons innervating the nRPC (Simons‐Weidenmaier et al., 2006). The source of this presynaptic depression is disputed, (i.e., depletion vs. feedback‐silencing of synaptic vesicles; Betz, 1970; Gover & Abrams, 2009; Gover et al., 2002; Zucker & Regehr, 2002); regardless of the exact mechanism, the result is that the nRPC receives less excitatory input with each stimulus iteration, thus lowering the probability and magnitude of activity in the nRPC and its motor efferents. Responding spontaneously recovers as a function of time elapsed between stimuli (Rankin et al., 2009).

Researchers typically only concern themselves with startle habituation for the steepest response decline during the first 2–10 trials, and treat it as a problem to control for rather than data to analyze for its own sake (Lane et al., 2013). Metrics of habituation also vary, and can include raw change (Y2‐Y1), percent change ([Y2–Y1]/Y1), or residualized change (e.g., regression slope, ((Y2‐Y1)/(X2‐X1)), and the changes measured can be overall or incremental (Lane et al., 2013; Llabre et al., 1991). Another point of contention is how to define initial startle magnitude. Not all participants habituate between the first and second trial; some respond the same, and others may even sensitize (respond more strongly). Sensitization is functionally distinct from habituation (Götz & Janik, 2011), and the extent of sensitization in these early trials may determine how best to define the beginning of a habituation block: as the response to trial 1, the response to trial 2, or the average response to the two trials (Lane et al., 2013; Valsamis & Schmid, 2011).

Research suggests that variability in startle habituation is relevant to the study of emotion regulation in general and anxiety in particular. There are three related parameters to potentially consider when modeling habituation: initial response magnitude, change in response magnitude, and asymptotic response magnitude (the response level at which future responses do not decrease significantly; Lane et al., 2013). Asymptotic and post‐asymptotic responding, while important in studies of emotion (Gorka et al., 2013; Grillon, 2002; Grillon et al., 2008; Grillon & Ameli, 1998; Michopoulos et al., 2015), requires special considerations when studied together with habituation because the number of trials needed for responses to reach asymptote varies (Thompson & Spencer, 1966), and it is difficult to tell how much the statistical distinction of asymptote relates to measurable changes in underlying biological kinetics (Rankin & Broster, 1992). Therefore, asymptotic responding will not be considered in great detail in this paper. However, both initial startle magnitude and habituation are related to the emotions of anxiety and fear. People with panic disorder have higher initial startle response magnitude than those without panic disorder (McTeague et al., 2011). Meanwhile, people who exhibit deficits in startle habituation score higher in anxiety sensitivity (i.e., fear of bodily concomitants of anxiety; Campbell et al., 2014; Reiss et al., 1986; Taylor et al., 2007), and have higher probability of anxiety disorders (Jovanovic et al., 2009; Lader & Wing, 1964). Since many researchers conflate these two measures by reporting average magnitude across trial blocks or session, the differential relationships between components of habituation and other factors cannot be seen in those studies. Investigating change in reactivity along with initial reactivity is a more sensitive way to evaluate those relationships (Burt & Obradović, 2013).

### 1.2 Initial value dependence

Based on the studies described above, one might reason that high initial magnitude and shallow habituation slope are related to one another via their mutual relation to anxiety. However, studies have yet to find that anxiety simultaneously predicts both high initial magnitude *and* reduced habituation (Campbell et al., 2014; McTeague & Lang, 2012). This seeming inconsistency harks back to a phenomenon that, like startle habituation, has been long recognized but perhaps not explored deeply enough: initial value dependence (IVD). IVD is a pattern in psychophysiology wherein extreme initial values constrain subsequent responses to avoid becoming more extreme, and to change more dramatically in the direction opposite the extremity. Classical understanding of IVD has focused on discrete change in a measure following some external manipulation (Benjamin, 1967), but IVD also holds for continuous and automatic processes (Jin, 1992). Dependence in terms of habituation means that the stronger initial responses are, the steeper the habituation slope.

IVD is pervasive in biological response measures, particularly in studies of autonomic and vascular activation measures (Benjamin, 1963, 1967; Hord et al., 1964; Jamieson, 1987; Lovallo & Zeiner, 1975; Messerli et al., 2015), but it has also been observed in studies of habituation of skin conductance responding (Germana, 1968) and autonomic recovery (i.e., blood pressure, stroke volume, and electrodermal activity; Myrtek & Foerster, 1986), as well as measures that are not directly related to autonomic activity (e.g., EEG; Block & Bridger, 1962; Myrtek & Foerster, 1986). The term *initial value dependence* has been used interchangeably with the *law of initial value* (Jin, 1992; Wilder, 1967), but these terms are not equivalent; the former is a broad pattern in physiological data (Jin, 1992; Raykov & Penev, 1996), whereas the latter denotes a homeostatic mechanism for IVD (i.e., regulatory control through negative feedback; Benjamin, 1967; Jamieson, 1993; Wilder, 1967). Other statistical and biological factors influence dependence as well (for a review, see Jamieson, 1993), but the factor of particular interest to the present study is trait physiological reactivity (Duffy, 1962;Jamieson, 1993, 1995; Jamieson & Howk, 1992), which is often higher in anxious people (Lang & McTeague, 2009). In more reactive individuals, high initial activity may not necessarily predict steeper habituation, resulting in IVD that is attenuated or even reversed from expectation.

The relationship between reactivity and IVD has not been found explicitly in terms of startle reflex habituation, but has been explored in prior work on autonomic responding. In some studies (Duffy, 1962; Scher et al., 1985) high baseline cardiac activity (heart rate, T wave amplitude) predicted exaggerated heart rate increases in response to a stress manipulation, rather than reductions in responding as might be expected. Myrtek and colleagues (Myrtek et al., 1977; Myrtek & Foster, 1986) explored IVD in a variety of autonomic and nonautonomic measures and manipulations, and likewise found reverse IVD effects in the majority of their measures after controlling for statistical artifact. Berntson et al. (1994) further posited that adherence to IVD may depend on the source of the initial variation, with stronger IVD effects found in their study when initial cardiac activity was orthostatically manipulated than when the initial activity was recorded at rest. To date, reverse‐IVD trends have still not been reliably observed when variation in initial values are manipulated, further suggesting that variation in IVD reflects trait‐ but not state‐level differences in physiological reactivity, and perhaps by extension trait anxiety but not contextual anxiety. Trait reactivity may thus be a particularly elegant explanation for between‐subject variation in IVD, based on theory (Myrtek & Foerster, 1986) as well as simulation research (Jamieson, 1993, 1995; Jamieson & Howk, 1992). Although no published work to date links anxiety to IVD in startle habituation, the links between startle reactivity and autonomic dysfunction (Mauss et al., 2003; Ruiz‐Padial et al., 2003), between IVD and physiological flexibility (Duffy, 1962; Jamieson, 1993; Scher et al., 1985) and between flexibility and emotion regulation (Waugh et al., 2008) imply that IVD in startle habituation may relate to emotion regulation as well.

**FIGURE 1:**
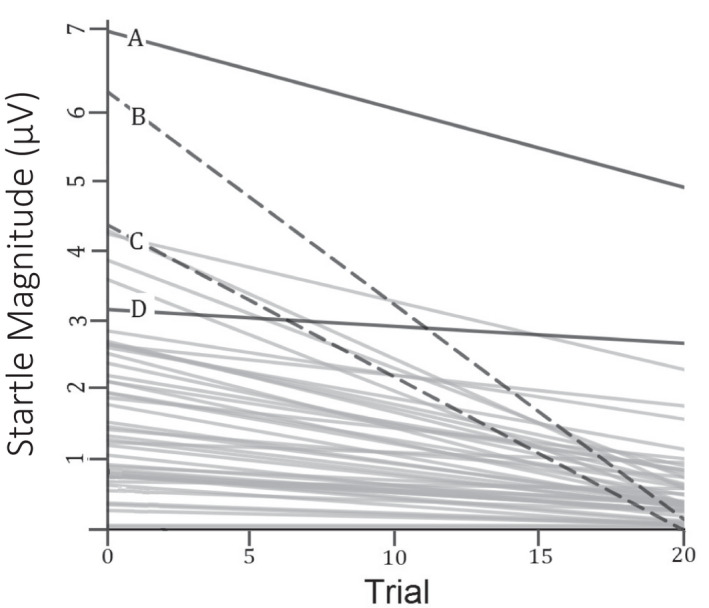
Hypothetical distribution of habituation curves, random IVD. Growth curves that illustrate a notable deviation from the formula are pictured in bold and labeled A, B, C, and D. solid lines A and D show attenuations of IVD, whereas dashed lines B and C show exaggerations of IVD

Classical studies of IVD involve a group‐based significance test: either the sample follows the law of initial value, or it does not (Jin, 1992). However, due to its between‐person variation, IVD may also be conceptualized as an individual differences variable. To illustrate, consider the distribution of growth curves pictured in Figure 1. The formula for predicting linear slope from initial value is linear slope = −(initial value)/12 + *e*, where *e* is a random distribution of values with a mean of 0 and a standard deviation of .07 (enough variation to be realistic without generating significant positive slopes). The asymptotic curvature of the growth curves, while not directly relevant to IVD, is modeled here as well, via a fixed equation of quad slope = −(linear slope)/40 to more accurately represent habituation kinetics. Notable deviations from the formula are pictured in bold and labeled A, B, C, and D. The solid lines A and D show attenuations of IVD; response decrements are not as steep as expected given initial value. A similar effect of diminished habituation of startle is caused by caffeine, a mildly anxiogenic drug (Benke et al., 2015; Schicatano & Blumenthal, 1998). Following prior logic, these relative deficits in habituation may signal poorer emotion regulation. Meanwhile, dotted lines B and C display a hypothetical exaggeration of IVD effects, with response decrements that are steeper than expected. EMG measurements are rectified, so a line crossing into negative values is not possible. This floor effect is important, because it constrains how much individual slopes can vary and, by extension, might reduce the amount of variability in slope explained by reactivity. If this is the case, then IVD may more accurately index physiological flexibility than does slope alone.

### 1.3 Study goals

The present study tested for relationships between startle habituation parameters and trait and contextual anxiety. Due to a lack of existing data exploring reactivity and IVD in the startle reflex, as well as the inconsistent relationship between anxiety and habituation when initial value is accounted for, the analyses were exploratory, and thus no specific hypotheses were made. Startle habituation was modeled using conditional growth curves, which allowed the simultaneous analysis of simple habituation kinetics along with higher‐order interactions with initial value measures, an anxiety manipulation, and trait anxiety measures. Analyses were thus able to partial out the potential roles of IVD and linear habituation slope as statistical metrics of biomarkers for emotion regulation in startle habituation.

## 2 METHOD

### 2.1 Participants

Participants (n = 39) were undergraduate psychology students at Wake Forest University, recruited over the course of a semester online through Sona Systems (Sona Systems, Ltd; Bethesda, MD) in exchange for course credit. The original aim was to recruit more than 60 participants, but time constraints due to academic graduation limited the number of participants that the study team was able to recruit. Before signing up for the study, participants completed the State Trait Anxiety Inventory‐Form Y, Trait subscale (Spielberger, 1983). To promote reliable and safe data collection, participants were excluded based on the following criteria: previous participation in a startle reflex study through the Blumenthal Psychophysiology Lab; the use of stimulant or depressant drugs (e.g., amphetamine, clonazepam, and alcohol) within the past 8 hours; hearing loss due to injury or illness; panic disorder, or seizure disorders. While relevant to the study of anxiety, panic disorder was listed as an exclusionary criterion in order to limit the likelihood of adverse reactions to the shock stimuli. Of those initially recruited, two were ineligible at screening, four declined participation partway through the experiment, one had lost data, and one had unreliable physiological data (high amount of noise artifact). This left the number of participants with complete data at 31; 15 of these were assigned to the control group, and 16 were assigned to the contextual anxiety (CA) group.

### 2.2 Stimuli

The startle stimulus was 100 dB broadband noise, with near instantaneous rise time and 50‐ms duration. Stimuli were created using Audacity 2.0 audio editing software (The Audacity Team; Pittsburgh, PA), and presented using SuperLab 5.0 (Cedrus Corporation; San Pedro, CA). The sound output of the computer was amplified using a PreSonus HP4 Amplifier and presented binaurally to participants via Sennheiser eH250 headphones. Stimuli were presented in two blocks of 20 stimuli, with intertrial intervals of 13 to 17 seconds.

The shock stimulus for the CA group was a single 100‐V, 3 mA burst of direct current, lasting 0.5 ms. Administration was controlled through a stimulator output channel in the AcqKnowledge 4.3 software package (Biopac Systems, Inc., Goleta, CA). The electrodes delivering the shock were 4‐mm silver chloride (Ag/AgCl) disc electrodes, filled with Signa Crème electrode cream to reduce impedance, and attached with removable adhesive collars. Anode and cathode were placed on a distal part of the left arm near the wrist, spaced approximately 1 cm apart, and plugged into a single‐channel stimulation amplifier using a programmable stimulator module STM100C (Biopac Systems, Inc., Goleta, CA).

### 2.3 Measures

#### 2.3.1 Physiological recording and processing

The study involved recording of eyeblinks and heart rate, the latter of which will not be discussed at length in this paper. Sensors consisted of five 4‐mm diameter Ag/AgCl electrodes (Gereonics Inc.), filled with conducting paste and attached with adhesive collars. In keeping with guidelines for eyeblink EMG (Blumenthal et al., 2005), electrode sites were cleaned with 70% isopropanol and then eyeblink sensors were placed 1 cm below the pupil, and 1 cm below the left lateral canthus. Two sensors were placed below the ribs, one on each side of the abdomen, for electrocardiography (ECG). An electrode on the sternum served as a ground for both EMG and ECG. Startle eyeblinks were recorded at a sampling rate of 1000 Hz using AcqKnowledge 4.3 software. EMG data were filtered with a 28–500 Hz passband, rectified, and then smoothed using a 5‐sample boxcar filter according to guidelines by Blumenthal et al. (2005). Blink peak magnitude within a window of 20–120 ms after startle stimulus onset was defined as two standard deviations above the mean EMG activity in the preceding 500 ms baseline period, and was quantified as the peak magnitude minus the baseline mean magnitude in microvolt*seconds (μV*s).

#### 2.3.2 Trait anxiety

Trait anxiety was measured using the State–Trait Anxiety Inventory for adults, Form Y, trait subscale (Spielberger, 1983), administered online to the introductory psychology student research pool at Wake Forest University through Qualtrics Survey Software. The questionnaire consisted of 20 questions, asking about the overall prevalence of different feelings of anxiety (e.g., “I felt nervous”), as well as reverse‐scored items (e.g., “I felt calm”), with item responses ranging from 1 to 4 in order of frequency. Scores have a possible range of 20 to 80, with higher scores representing greater trait anxiety. The questionnaire was reliable, with Cronbach's alpha at .91.

#### 2.3.3 State anxiety

State anxiety was measured using the six‐item short form of the State Trait Anxiety Inventory‐Form Y, state subscale (Spielberger et al., 1983), developed by Marteau and Bekker (1992). Respondents gave ratings from 1 to 4 in order of extremity for current anxious (or anxiety‐absent) feelings. Scores were multiplied by 20/6 to be comparable with the original scale, according to guidelines by the scale authors. Possible scores range from 20 to 80, with higher scores representing greater acute distress. Reliability for the state anxiety measure in the present sample was lower than for the trait anxiety measure (i.e., with an average Cronbach's alpha across three test administrations of .69), probably due to the lower number of items (Marteau & Bekker, 1992; Tavakol & Dennick, 2011).

### 2.4 Procedure

Part 1 of the experiment was the same for both control and contextual anxiety groups. Participants filled out the first of two consent forms, followed by a health history questionnaire to screen for exclusion criteria, and the first state anxiety questionnaire. After this, the EMG and ECG electrodes were placed, and the participant sat quietly for a 330‐second heart rate recording. Next, the experimenter placed headphones on the participant and, after determining that the EMG recording noise was suitably low (below .2 μV), delivered the first block of 20 startle stimuli. Immediately after the startle block, the experimenter removed the headphones and started the second 330‐sec heart rate recording, followed by another state anxiety questionnaire.

Participants then gave informed consent for the second part of the study. Only participants in the CA group were informed about the shock. For the contextual anxiety condition, the experimenter placed shock electrodes on the underside of the participant's left arm near the wrist, and explained that at some point during the session the participant may receive a brief shock. The experimenter then administered a sample shock, followed by the last state anxiety measure, and then the next block of 20 startle stimuli. During the startle block, participants did not receive another shock, based on the reasoning that mere anticipation of shock is sufficient to induce anxiety (Schmitz & Grillon, 2012). After the second startle block was finished, the experimenter removed the shock electrodes, and then again measured heart rate for 330 sec. For the control group, part 2 was equal in length to that of the contextual anxiety group, with equal spacing of the tests. During the time that participants would have had the shock electrodes placed and tested, control participants were instead told to wait quietly in the room until the experimenter returned (2 minutes).

### 2.5 Analysis

#### 2.5.1 Defining initial value

All analyses were conducted using IBM SPSS Statistics for Windows, Version 25.0 (IBM Corp., Armonk, NY). Studies modeling habituation have defined initial value as magnitude at first trial (Casto & Printz, 1990), magnitude at second trial (Lane et al., 2013), or the average magnitude of the first and second trial (Valsamis & Schmid, 2011). The rationale for determining which metric to use depends on study goals, as well as considerations of how sensitization and habituation may separately impact response magnitude between trials 1 and 2. If magnitude decreases dramatically for every subject between trials 1 and 2, then habituation can be assumed to begin relatively uninterrupted by sensitization from the beginning of trial 1, and it may not be necessary to partial out sensitization effects from habituation estimates. If at least some subjects appear to sensitize, then additional considerations are needed. One potential solution is to define initial value for each individual as the highest level of the response before the response magnitude starts to decrease (i.e., at trial 2 or perhaps later). This would be inappropriate however, as it presumes that the process of habituation does not begin until sensitization ends, when in reality these learning mechanisms occur independently and their influences sum to equal a net effect on behavior. Defining initial magnitude as the average magnitude across the first two or more trials would also be problematic, as it may bias the kinetics of sensitizing subjects toward higher initial value and greater habituation than if they did not sensitize (e.g., in Meincke et al., 2004). To inform the study definition of initial magnitude, we examined individual startle eyeblink responses to trials 1 and 2 visually, and then analyzed those responses separately by block as a function of trial number, Group, and Trait Anxiety using general linear mixed modeling.

#### 2.5.2 Within‐ and between‐participant differences in state anxiety

We conducted a mixed ANOVA to test for changes in state anxiety between measurements, as well as the effect of treatment group. The interaction effect between state anxiety (SA) assessment time‐point and Group, tested by a priori contrast of measurements before and after the shock treatment (or lack thereof), served as a manipulation check for the shock treatment.

#### 2.5.3 Within‐ and between‐participant differences in startle habituation

Due to the design of the experiment, we anticipated that study variables would have a 3‐level nested structure, with Trials (level 1) nested within Blocks (level 2), and Blocks in turn nested within Participants (level 3). To verify if multilevel modeling was necessary and appropriate, we ran an initial null multilevel model with nesting variables specified as Block at level 2 and Participant at level 3. Intraclass correlation coefficients (ICCs) were calculated using the three levels' respective variance components, to examine the data for statistical nesting at level 2 and level 3. Variables were then added to the null model for model building and analysis. To estimate nonlinear trends of change at level 1, two trial‐level variables were included in the model in a manner similar to prior work (Lane et al., 2013): Trial Number (for linear change), and Squared Trial Number (for quadratic change). Trial Number was centered and then squared to produce the Squared Trial Number variable, so that intercept values would not be redundant in meaning with the raw Initial Value variable, and additionally with the intent to minimize collinearity between Trial Number and Squared Trial Number, as the latter was not expected to be as relevant as the former for variables of interest (for background see Biesanz et al., 2004; Mirman et al., 2008). The resulting values are represented in a supplement.

Ideally, the effect of contextual anxiety could be gauged by a fixed interaction effect of Group and Block (since the shock treatment occurred only for Group 2 in Block 2). However, the variable Block only had two levels, which precluded including both a random intercept and slope for block, as there would need to be at least one additional level relative to the number of random effects. To circumvent this issue, the shock manipulation was analyzed as a block‐level dummy variable with values indicating either that shock occurred (= .5) or that it did not occur (= − .5).

#### 2.5.4 Initial value dependence

We modeled startle habituation and its moderators using hierarchical linear modeling. A visual inspection of the relationships between startle magnitude and predictor variables suggested that the data satisfied the linearity assumption, and a visual inspection of model residuals suggested that the residuals were homoscedastic and normally distributed. Full maximum likelihood estimation was used for the model building process, and restricted maximum likelihood estimation was used for the main analysis due to the small sample size. The level 1 model tested the effects of Trial and Squared Trial number on trial‐by‐trial startle magnitude. The level 2 model tested the effects of blockwise Initial Value, the Contextual Anxiety condition, and the interaction between Initial Value and Contextual Anxiety, to serve as a comparison to a counterpart higher‐order interaction effect subbing Contextual Anxiety with Trait Anxiety. Lastly, the level 3 model tested main effects and cross‐level interaction effects of Trait Anxiety. Steps for building the models are represented as formulas in the appendix of this paper.

Trial number and squared trial number were additionally specified as repeated effects nested within blocks, which was an improvement over the level 1 model with random intercepts only (Akaike's Information Criterion [AIC] = 4592 for random intercept only, 4506 for random intercepts + repeated measures). Various covariance structures used for repeated measures in mixed models (for a review, see Kincaid, 2005) were then tested on the full 3‐level model for fit relative to the SPSS default diagonal method. The model would not run successfully with an unstructured covariance matrix, probably due to the sample's relatively small size and large number of repeated measures (Skene & Kenward, 2010). Alternative structures were tested on the full model for final selection based on model fit (AIC), model convergence, and positive definite final Hessian matrix. Of these, the best comparative fit that converged properly with 100,000 iterations (AIC = 4393 compared to 4396 with the diagonal structure) came from the autoregressive heterogeneous structure (ARH1), which assumes unequal variances between observations and cross‐measurement correlations that decay over time (Wolfinger, 1996). Block‐level variables of Initial Value (IV) and Shock condition were initially specified as random effects nested within individuals. The model did not converge properly with Shock specified as a random effect however, so the final model was run with only IV specified as a random effect in level 2.

## 3 RESULTS

### 3.1 Descriptive statistics for startle magnitude

**FIGURE 2:**
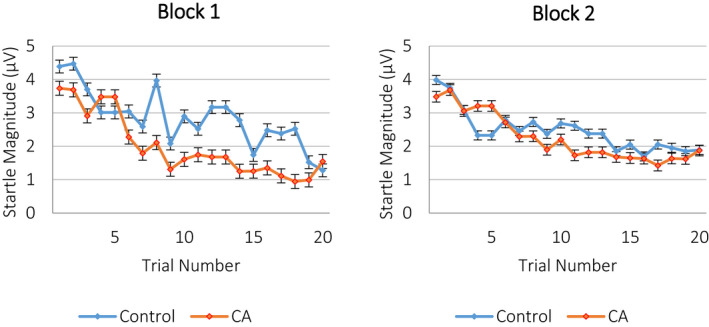
Startle magnitude by group, Block, and trial. Average startle magnitude at each trial, split by group and block. Error bars represent standard error. CA, Contextual anxiety group

Startle magnitude was positively skewed (skewness = 1.03; SE = .38), but within acceptable guidelines of ±2 (Tabachnick et al., 2001). Average startle magnitude by block and trial is represented in Figure 2. Startle magnitude at trials 1 and 2 are represented in Table 1 as means split by CA group, block, and trait anxiety (25th and 75th percentile). One participant had missing data for trial 2 of block 1, which affected the degrees of freedom for subsequent analyses.

**TABLE 1:** Startle magnitude (mag) at trial, by group and trait anxiety (TA)

	By group		By TA	
	Control	CA	Low	High
Block 1 Trial 1 mag	4.39	3.74	2.87	5.04
Block 1 Trial 2 mag	4.47	3.69	2.91	5.08
Block 2 Trial 1 mag	3.98	3.48	2.53	4.88
Block 2 Trial 2 mag	3.75	3.68	2.50	4.60

*Note*: Startle magnitude is expressed in microvolts. CA, Contextual Anxiety. Low and high TA are represented as 25th and 75th percentile scores, respectively.

**FIGURE 3:**
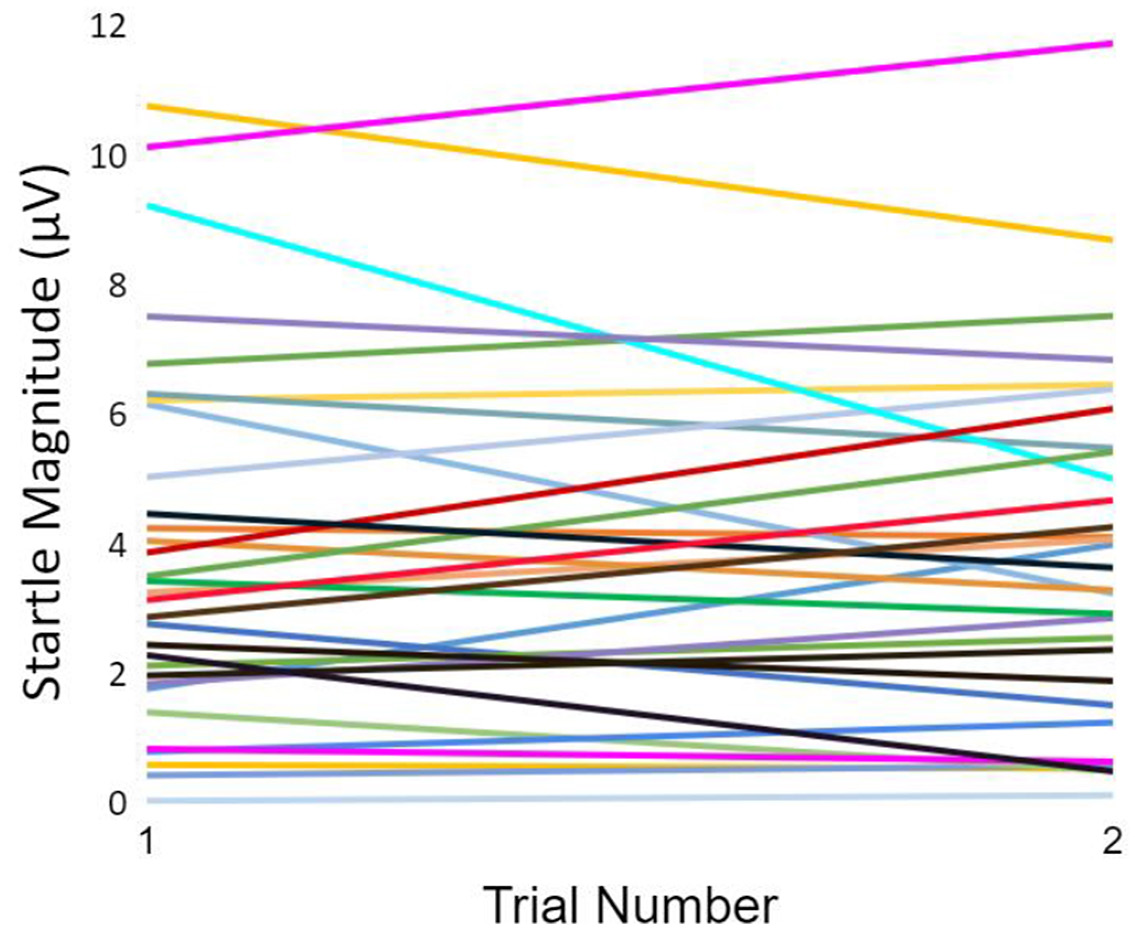
Initial startle magnitude of individual participants. Startle response magnitude on trials 1 and 2 are represented for each participant, to illustrate patterns of initial habituation and sensitization in the sample

**FIGURE 4:**
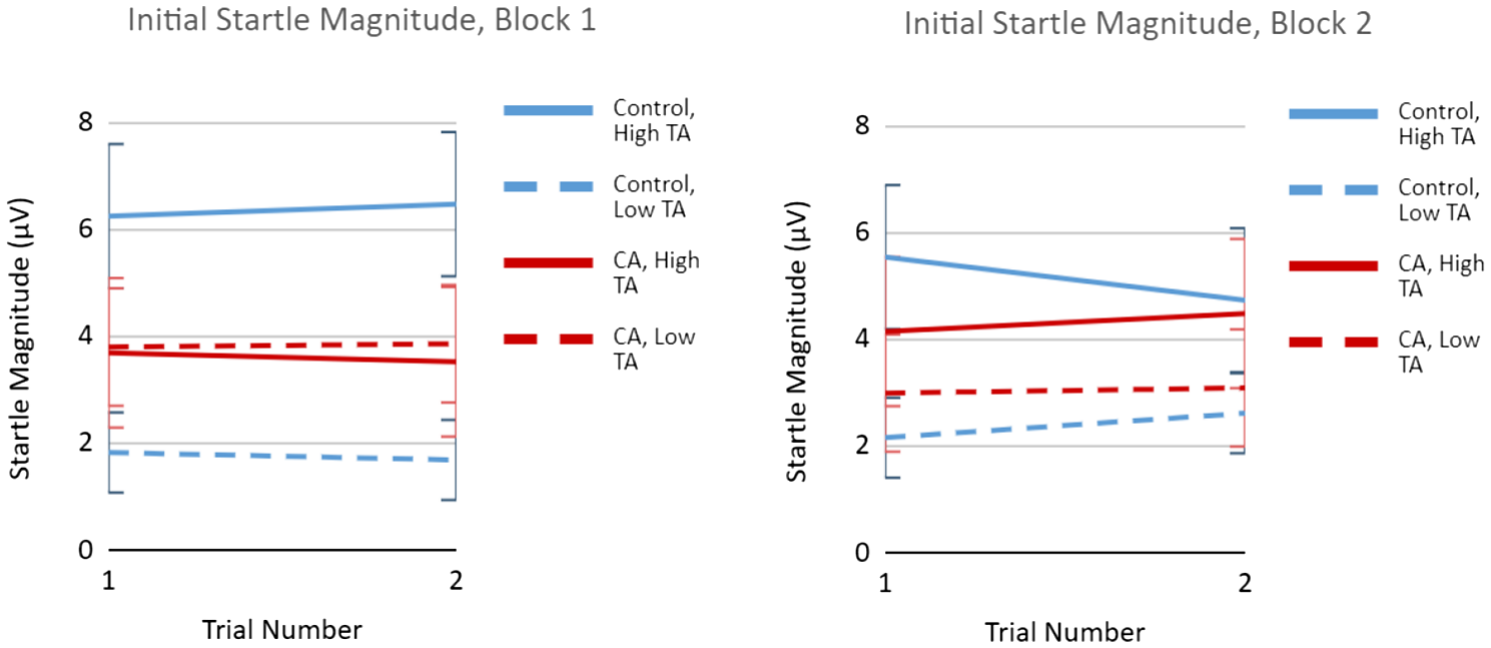
Initial startle magnitude by group and trait anxiety (TA). Startle response magnitude on control trials 1 and 2 by treatment group (control, CA) and trait anxiety (low TA = 25th percentile trait anxiety scores, high TA = 75th percentile of trait anxiety scores). Error bars represent 95% standard error confidence intervals. CA, Contextual anxiety group. Low and high TA are represented as 25th and 75th percentile scores respectively

A visual inspection of individual responses suggested that some participants sensitized, some habituated, and some stayed the same in magnitude between these trials (see Figure 3). With this heterogeneity in mind, and with the intent to minimize the effects of sensitization on initial magnitude and habituation estimates (e.g., as illustrated by Meincke et al., 2004), initial response value was defined as the startle magnitude at trial 1 in later multilevel analyses. Overall, startle magnitude did not change significantly from trial 1 to trial 2 in either block (Block 1 *β* = −.01, F(1,26) = .03, *p* = .854; Block 2 *β* = −.01, F(1,27) = 1.16, *p* = .955). Moreover, an a priori *t* test showed that initial magnitude was not different between block 1 and block 2 (Cohen's d = .05, t[30] = −0.75, *p* = .459). In both blocks, anxious participants had higher initial startle magnitude (Block 1 *β* = .38, F(1,26) = 6.30, *p* < .05; Block 2 *β* = .48, F(1,27) = 4.33, *p* < .005). Also of note, in both blocks the association between trait anxiety and startle magnitude was more strongly positive for the control group than for the CA group, based on a significant group*TA interaction (Block 1 *β* = −.47, F(1,26) = 8.97, *p* < .01; Block 2 *β* = −.38, F(1,27) = 6.78, *p* < .05). Marginal means of initial startle magnitude by group and trait anxiety level (25th and 75th percentiles for low and high TA groups, respectively) are presented in Figure 4.

### 3.2 Within‐ and between‐participant effects on state anxiety (SA)

**FIGURE 5:**
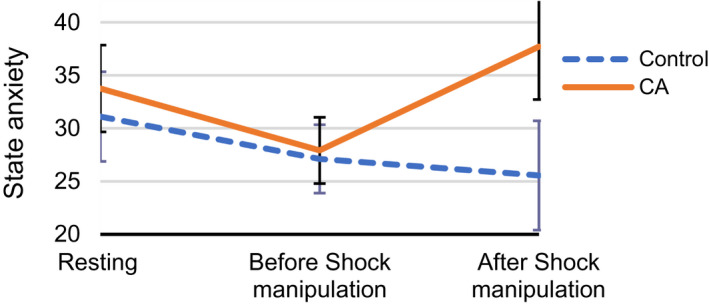
State anxiety by measurement time and group. Results show an increase in state anxiety in the contextual anxiety (CA) group between the second and third state anxiety measurement, due to the shock manipulation. Error bars represent 95% confidence intervals. CA, contextual anxiety group

Results of mixed ANOVA are in Table 2. The main effect of Group was significant (*β* = .29, F(1,27) = 4.52, *p* < .05). State anxiety did not change overall (*β* = −.03, F[2,54] = 1.83, *p* = .168), but there was a significant interaction between Time and Group (*β* = .22, F[2,54] = 10.58, *p* <. 001). The manipulation check, a contrast between the second and third state anxiety measurement, suggested that the shock manipulation increased state anxiety more than the control condition did (Cohen's d = 1.27, F[1,29] = 14.46, *p* < .01). Marginal means from this contrast are illustrated in Figure 5. Groupwise test–retest correlations for state anxiety measurements are presented in Table 3, comparing the first and second administration (before the anxiety manipulation) and the second and third administration (before and after the anxiety manipulation). Both comparisons were significant in the control group, and marginally significant in the Contextual Anxiety group.

**TABLE 2:** Within‐ and between‐participant effects on state anxiety

	Pillai's trace	*df*1	*df*2	*F*	*Sig*
Within‐person					
Time	.164	2	54	1.83	.171
Between‐group					
Intercept	–	1	27	17.00	<.001***
Group	–	1	27	4.52	.043*
Trait anxiety	–	1	27	1.25	.274
Interaction effects					
Time*Group	.364	2	54	10.60	<.001***
(SA1 vs. SA2 + SA3)	–	1	27	4.73	.038*
(SA2 vs. SA3)	–	1	27	14.50	.001**

*Note*: Time = measurement occasion (at rest = 1, after CA manipulation = 2); Sig = *p* value.

* *p* = .05

** *p* < .01

*** *p* < .001. *p* values for within‐person effects were calculated with sphericity assumed.

**TABLE 3:** Test–retest state anxiety correlations, by group

	Control		CA	
	r	Sig	r	Sig
SA1 vs. SA2	.773	<.001***	.492	.053
SA2 vs. SA3	.707	.003**	.468	.068

Abbreviations: SA, measurement occasion; r, correlation; Sig, p value.

** *p* < .01

*** *p* < .001.

### 3.3 Modeling startle habituation kinetics

Intraclass correlation coefficients (ICCs) were calculated based on variance components for the null model (residual = 2.66, level 1 intercept = .48, and level 2 intercept = 2.88). There are two ICCs in 3‐level designs: the level 2 ICC equals proportion of total variance in startle magnitude attributed to nesting of level 1 within levels 2 and 3; and the level 3 ICC equals proportion of total variance attributed to nesting of level 2 within level 3 (Hedges et al., 2012). The level 2 ICC was .48 / (2.66 + .48 + 2.88) = .08, or 8% of variance. The level 3 ICC was 2.88 / 2.66 + .48 + 2.88) = .48, or 48% of variance. For both ICCs, a number higher than 5% indicates that hierarchical linear modeling is recommended to avoid violating the assumption of independence (Tabachnick et al., 2001).

Standardized estimates of fixed effects and variance components are represented in Table 4. All models showed improved fit (lower deviance) compared to the null model (null model deviance = 4799), and the addition of each of the three levels improved the fit of the model before it, although not significantly so in the case of the three‐level model (Null model to level 1 model χ2(22) = 358.47, *p* < .001; level 1 model to level 2 model χ2(10) = 122.20, *p* < .001; level 2 model to level 3 model χ2(6) = 9.00, *p* = .174). Despite its nonsignificant improvement of fit over the 2‐level model, the full 3‐level model was retained as the final model due to the descriptive reduction in deviance and due to the theoretical importance of trait anxiety for study variables.

**TABLE 4:** Effects of block‐ and participant‐level factors on startle habituation

	Trial model		Block model		Participant model	
	B (SE)	Sig	B (SE)	Sig	B (SE)	Sig
Intercept	2139.37 (315.74)	<.001***	2140.19 (212.24)	<.001***	2120.79 (218.83)	<.001***
Level 1						
Trial	−.103.91 (8.71)	<.001***	−108.93 (7.58)	<.001***	−115.07 (7.93)	<.001***
Trial^2^	6.69 (1.62)	<.001***	6.99 (1.42)	<.001***	6.86 (1.49)	<.001***
Level 2						
IV	–	–	355.42 (63.93)	<.001***	333.17 (68.25)	<.001***
Shock	–	–	351.59 (222.65)	.120	333.62 (224.15)	.143
IV*Shock	–	–	64.39 (99.49)	.521	76.71 (101.09)	.452
Level 3						
TA	–	–	–	–	23.39 (24.51)	.347
Interactions						
IV*Trial	–	–	−26.27 (2.62)	<.001***	−27.83 (2.99)	<.001***
IV*Trial^2^	–	–	1.87 (.49)	<.001***	2.03 (.56)	<.001***
Shock*Trial	–	–	−6.42 (17.38)	.712	−2.06 (17.45)	.906
Shock*Trial^2^	–	–	2.25 (3.23)	.486	2.21 (3.27)	.501
IV*Shock*Trial	–	–	−14.37 (6.95)	.040*	−11.28 (7.18)	.117
IV*Shock*Trial^2^	–	–	−.20 (1.29)	.879	−.41 (1.35)	.764
TA*Trial	–	–	–	–	−.89 (.90)	.326
TA*Trial^2^	–	–	–	–	−.21 (.17)	.218
TA*IV	–	–	–	–	4.71 (7.20)	.518
TA*IV*Trial	–	–	–	–	.65 (.26)	.013**
TA*IV*Trial^2^	–	–	–	–	.002 (.05)	.969
Model fit						
(Deviance)	4440		4318		4393	

*Note*: Startle magnitude is expressed in nanovolt * seconds (nV*s) to follow APA reporting guidelines. IV=Initial startle magnitude; TA = trait anxiety; *e*, *r*_0_, and *r*_1_ are error terms. Standard errors are in parenthesis. Deviance is expressed as −2 Log likelihood, where smaller values indicate relatively better fit.

* Significant at *p* = .05

** Significant at *p* = .01

*** Significant at *p* = .001.

In the full model, Level 1 parameter estimates for trial and squared trial number were both significant (*β* = −.27, t(285) = −14.84, *p* < .001; *β* = .08, t(277) = 4.71, *p* < .001, respectively), indicating significant linear and curvilinear components to startle habituation. As expected, a significant effect of initial value (*β* = .49, t[37] = 5.22, *p* < .001), indicated that initial startle magnitude predicted average startle magnitude. Both linear slope and quadratic slope were moderated by initial value (*β* = −.20, t(288) = −9.59, *p* < .001; *β* = .07, t(283) = 3.72, *p* < .001, respectively). The former moderating effect (IVD) was in the expected direction, and indicated that initial value significantly and negatively predicted average block‐level slope. The significant positive interaction effect between initial value and squared trial number indicated that higher initial value predicted a more positive (i.e., stronger) curvilinear trend.

Trait anxiety did not impact linear slope (*β* = −.02, t[284] = −.10, *p* = .320), but moderated IVD, in a direction suggesting attenuation of IVD effects as TA increased (*β* = .04, t[287] = 2.52, *p* < .05). The shock manipulation in the full model did not impact overall startle magnitude (*β* = .07, t[59] = 1.54, *p* = .129), linear habituation slope (*β* = −.002, t(285) = −.10, *p* = .921), or IVD (*β* = −.03, t(285) = −1.58, *p* = .116). However, the effect of the Shock*Trial*IV interaction was significant in the 2‐level model, in a direction suggesting stronger IVD effects when participants were being threatened with shock (*β* = −.04, t[273] = −2.07, *p* < .05).

**FIGURE 6:**
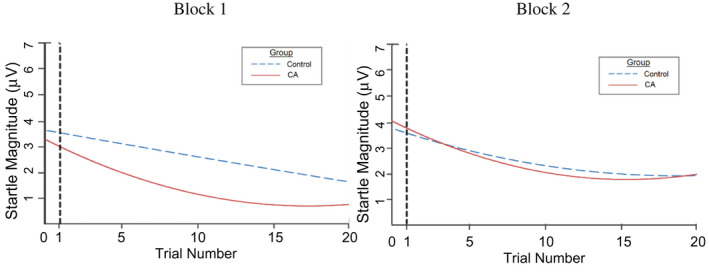
Habituation growth curves by group, separated by blocks. Marginal habituation growth curves split by block illustrate group differences (control vs. CA group) in startle kinetics. CA, contextual anxiety group

**FIGURE 7:**
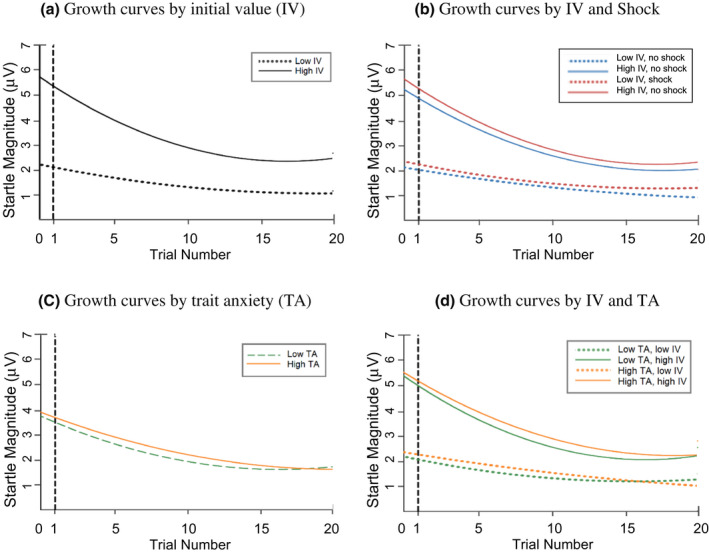
(a–d) Habituation growth curves, averaged across blocks. Growth curves illustrate marginal habituation kinetics based on initial value (IV; Figure 7a), initial value and shock (figure 7b), trait anxiety (TA; figure 7c), and initial value and trait anxiety (figure 7d). Low and high IV and TA are presented as 25th and 75th percentile scores, respectively

Regression equations illustrating relevant sample differences in habituation kinetics are represented in various forms in Figures 6 and 7 through 7D. Slopes represent marginal effects, as in unique differences in startle habituation patterns remaining after accounting for all other variables. Low and high groups for continuous scales are constructed by splitting the distribution of scores into 25th and 75th percentiles, respectively. Figure 6 further supports group differences in startle kinetics in both Block 1 and Block 2. Moreover, the graphs illustrating marginal habituation kinetics based on trait anxiety, initial value, and the IV*TA interaction show that some groups differ markedly (e.g., by initial value in Figure 7), while others do not (e.g., based on trait anxiety in Figure 7c). Lastly, the crossing of lines in graph 7D but not 7B supports significant interaction effects of TA, but not shock, on IVD.

## 4 DISCUSSION

The present study explored how trait and contextual anxiety relate to habituation kinetics of the startle eyeblink response. Trait‐anxious participants did not differ in how much they habituated overall, but the degree of their habituation depended less strongly on initial startle magnitude than did that of their less anxious counterparts. The results provide modest support that variation in initial value dependence in startle habituation reflects differences in physiological and emotional reactivity, consistent with prior research (Scher et al., 1985) and theory (Duffy, 1962; Jamieson, 1993, 1995). Moreover, the results suggest that trait anxiety may predict IVD even when its relationship to habituation is less consistent. These results highlight potential benefits of studying emotional kinetics in a more nuanced fashion than by session averages alone.

Prior research suggests that measures of phasic physiology are valuable in the study of emotion. Differences in patterns of habituation are proposed to relate broadly to emotion regulation, with trait and clinical anxiety theoretically predicting deficits in habituation in a variety of physiological measures. Some results in prior studies are in line with this prediction (Campbell et al., 2014; Lader & Wing, 1964), while others suggest no effect or even opposite effects, with more reactive participants habituating more dramatically (Lader, 1964; Lane et al., 2013). One reason for inconsistent findings in the literature might be the high correlation between initial response magnitude and subsequent change in activity, which confounds an easy interpretation of what slope alone means in terms of physiological flexibility (Campbell et al., 2014; Lane et al., 2013). Initial value dependence may be a relevant phenomenon in itself; when the rate of decline toward baseline following an arousing event is not commensurate with initial response magnitude, this could reflect deficits in recovery from stress. It may thus be useful to consider that the biology of IVD involves specific neurological circuits related to emotion regulation (e.g., prefrontal cortex), in addition to the spatially diffuse homeostatic feedback loops typically implicated (Jin, 1992).

The significant effects found when analyzing IVD suggest that IVD, and not linear slope, is impacted by trait anxiety. Given the well‐supported relationship between anxiety and physiological reactivity (Benke et al., 2015; Seligowski et al., 2016; Yang & Friedman, 2017), these results are in line with theoretical and empirical work supporting the role of reactivity in between‐person variation in IVD (Duffy, 1962; Jamieson, 1992; Jamieson, 1993; Scher et al., 1985). These results also further strengthen the rationale for looking at startle habituation in a regression‐based framework, rather than simply pooling across trials within a block or session. While the statistical properties of habituation and IVD may be interesting to some on a basic science level, it is important not to lose sight of the reason why they are important in emotional psychophysiology. While initial value dependence is not the primary focus of this paper, its relation to trait anxiety in the present study may provide information about how to properly measure emotional flexibility. Physiological response change, and by extension the emotional change it represents, may need to be analyzed not in raw form but as part of a larger context of emotional responding.

While results support the role of trait emotional reactivity in IVD, the effect of contextual anxiety is less clear. In the three‐level model, the threat of shock did not impact overall startle magnitude, habituation slope, or IVD, despite increased self‐reported state anxiety. This finding differed from the significant effects of shock seen in the two‐level model that did not include trait anxiety, which implies that the explanatory power of trait anxiety both overlapped with that of contextual anxiety, and subsumed it. These disparate findings may mean that the shock manipulation's effect on anxiety was not consistent enough across subjects for downstream physiological effects to be properly detected. Alternatively, it is possible that individual variation in IVD is more sensitive to trait‐level than context‐level differences in physiological responding, as suggested by Scher et al. (1985). More research is needed.

### 4.1 Limitations

The present study had a number of limitations worth discussing, one being the test–retest design of the anxiety manipulation. Mixed between‐within‐participant studies are excellent at reducing error variability and optimizing power in small samples (Howell, 2012), and studies of startle responding are no exception (Cook et al., 1991). However, making inferences about startle habituation kinetics in two‐block designs comes with some special considerations. Full recovery of baseline reactivity between blocks can take anywhere from hours to days (Rankin et al., 2009), so when blocks are presented in relatively quick succession, initial reactivity does not completely “reset” to baseline levels. However, we tested for this recovery and found it to be sufficient, such that startle reactivity at the beginning of the second block was not significantly different from that at the beginning of the first block (see Table 1).

Additionally, prior research suggests that exposure to the first presentation block of stimuli can potentiate the effects of habituation in the second block, with faster and more complete reductions in startle responding in block 2 (Rankin, 2009; Rankin et al., 2009). A comparison of confidence intervals for the effect of linear habituation between Block 1 and Block 2 suggested that linear slope, like reactivity, did not differ significantly between blocks in the present sample. Regardless, there may still be differences between presentation blocks that confound variance attributable to the shock manipulation. These effects could have been partialled out with a fully counterbalanced design, featuring four different conditions: startle alone for both blocks; startle alone in block 1, startle + shock in block 2; startle + shock in block 1, startle alone in block 2; and startle + shock in both blocks. This design would ideally have needed complete data from far more participants to have sufficient power, which was not feasible in our lab at the time, but may still be worthwhile as a future direction.

The definition of initial value as the magnitude of response on trial 1 was a well‐reasoned choice, but it was predicated on a set of assumptions about sensitization and habituation that could benefit from future exploration. Future work focusing on startle habituation may find interesting information about psychologically relevant variables for who tends to sensitize and who tends to habituate from trial 1 to 2. Startle stimuli are not merely a probe of distress but a cause of it; loud noises are aversive, so temporary sensitization after the first trial could occur due to individual differences in fear learning, mediated by bidirectional projections between the amygdala and the nucleus reticularis pontis caudalis (nRPC). Differences in the strength of these connections and in fear learning may thus distinguish habituators from sensitizers in these early trials, and may therefore be worth exploring.

An additional question left unanswered in the present study is whether the effect of the contextual anxiety condition on IVD depended on differences in trait anxiety. Due to modeling constraints we were unable to test for any CA*TA interactions, but it may be a good future point of inquiry. The fact that the Shock*IV*Trial interaction was significant in the two‐level model before adding main effects and interactions with TA may suggest that the effect of contextual anxiety on IVD could be qualified by an interaction between contextual and trait anxiety, further underscoring the need for future work to examine state and trait influences on IVD.

One puzzling finding that needs further discussion is the apparent group differences in startle kinetics in both blocks of stimuli. Specifically, the association between trait anxiety and startle magnitude was more strongly positive for the control group than for the CA group. This interaction seems unexpected given random assignment of the CA condition and equal treatment of the groups in block 1. There are a number of possible explanations for this finding. One possibility is a sampling error, wherein the CA condition was assigned to participants who were higher in reactivity. While not impossible, this explanation seems unlikely given that CA and control participants did not differ in other parameters of startle, in trait anxiety, or in pre‐manipulation state anxiety. Another potential issue may be subtle differences in experimenter behavior due to having advance knowledge of each participant's assigned condition. The experimenter was mindful of the dangers of experimenter bias, and it is possible that in an effort to avoid biasing the CA group toward greater distress, the experimenter inadvertently made CA participants feel more comfortable instead of equally as comfortable as control participants. Lastly, it is possible that these results are merely spurious. Regardless, more research is needed to be certain why these results occurred the way that they did.

Another point to discuss is one of psychometrics. The present study's conclusions about the psychophysiology of dispositional anxiety are based on assumptions that the State–Trait Anxiety Inventory (STAI) measures anxiety as a construct. There is evidence that questions the construct validity of the STAI (Balsamo et al., 2013; Ramanaiah et al., 1983), and some authors claim that the trait measure more likely indexes general negative affect as opposed to anxiety specifically (Balsamo et al., 2013). If this is the case, then the present study's distinct results for trait (but not contextual) anxiety might have more to do with the assessment of different constructs than state versus trait measures of the same construct. More research is needed to clarify the distinctions among facets of negative affect, which is complex and multidimensional.

Similarly, one should exercise caution in ascribing the study's effects to principles of habituation more broadly. Elicitation of startle requires presentation of an intense stimulus which may be perceived as aversive, especially by people who are anxious to start with. It is possible that the effects of anxiety on IVD within startle habituation may not generalize to habituation of other reflexes, so more research is needed before making such broad claims.

Lastly, as with any small study, our study cannot be discussed without acknowledging issues related to power. A low number of participants can yield sufficient power to detect effects in startle research (Kedzior et al., 2016; Larson et al., 2005), especially with the rigorous steps that our research team took to mitigate acoustic and electrical noise (e.g., careful handling of electrodes, measuring the EMG signal noise level before recording, etc.). However, it is certainly possible that the failure to find significant results of individual‐level analyses in this study (i.e., the effects of both contextual anxiety and trait anxiety on startle habituation, and the effect of contextual anxiety on IVD) occurred due to low power instead of the actual absence of an effect. Hence, our arguments that the study results support some psychophysiologically relevant dissociations between state and trait anxiety, or between startle habituation slope and IVD, are suggestive, but do merit some healthy skepticism. More and higher‐powered work will be needed to delve into these relationships further.

## 5 CONCLUSION

In sum, the present study explored the impact of contextual and trait anxiety on startle habituation kinetics, and particularly on initial value dependence. Results suggest that trait anxiety predicts the strength of the relationship between initial value and degree of habituation—and not merely degree of habituation per se. In light of this finding, it is recommended that future research modeling the unique relationship between physiological change and psychological variables account for initial value as a covariate. Due to high correlation between initial value and linear slope, the choice to model both IVD and slope may admittedly reduce the unique explanatory variance in change scores, and with it the chance of finding significant relationships. However, the prevalence of dependence in biological change measures should not be ignored. Moreover, simultaneous modeling of both change and IVD could be useful in determining the conditions under which linear slope or IVD serve as optimal phasic markers for anxiety. Future studies might then test whether IVD or slopes are more important for other phasic measures (e.g., heart rate change, electrodermal response), or predicted by other measures related to emotion regulation (e.g., negative affectivity), which may have practical implications in the understanding and treatment of emotional problems.

## AUTHOR CONTRIBUTIONS

**Jules Alex Faunce:** Conceptualization; data curation; formal analysis; investigation; methodology; project administration; visualization; writing – original draft; writing – review and editing. **Terry D. Blumenthal:** Conceptualization; data curation; project administration; resources; software; supervision; writing – original draft; writing – review and editing. **Christian E. Waugh:** Conceptualization; writing – original draft; writing – review and editing.

## CONFLICT OF INTEREST

The authors have no affiliations with or involvement in any organization or entity with any direct financial interest (such as honoraria; educational grants; participation in speakers' bureaus; membership, employment, consultancies, stock ownership, or other equity interest; and expert testimony or patent‐licensing arrangements), or nonfinancial interest (such as personal or professional relationships, affiliations, knowledge or beliefs) in the subject matter or materials discussed in this manuscript.

## Supporting information


**TABLE S1** Coding of the time variable for level 1 slopes

## APPENDIX A

### A.1

Level 1 (Trial)

${\text{MAGNITUDE}}_{\mathrm{ijk}}=\pi_{0\mathrm{jk}}+\pi_{1\mathrm{jk}}*\left({\text{TRIAL}}_{\mathrm{ijk}}\right)+\pi_{2\mathrm{jk}}*\left({{\text{TRIAL}}^{2}}_{\mathrm{ijk}}\right)+e_{\mathrm{ijk}},$

where MAGNITUDE_*ijk*_ is startle magnitude at trial *i*, block *j*, individual *k*; *π*_0*jk*_ is the intercept of the equation predicting startle magnitude from trial; *π*_1*jk*_ is linear slope of change in magnitude for block *j*, individual *k*; *π*_2*jk*_ is the quadratic slope of change in magnitude for block *j*, individual *k*; and *e*_*ijk*_ is the level 1 residual.

Level 2 (Block)

$\pi_{0\mathrm{jk}}=\beta_{00k}+\beta_{01k}*\left({\mathrm{IV}}_{\mathrm{jk}}\right)+\beta_{02k}*\left({\mathrm{CA}}_{\mathrm{jk}}\right)+\beta_{03k}*\left(\mathrm{IV}*{\mathrm{CA}}_{\mathrm{jk}}\right)+r_{0\mathrm{jk}}.$

$\pi_{1\mathrm{jk}}=\beta_{10k}+\beta_{11k}*\left({\mathrm{IV}}_{\mathrm{jk}}\right)+\beta_{12k}*\left({\mathrm{CA}}_{\mathrm{jk}}\right)+\beta_{13k}*\left(\mathrm{IV}*{\mathrm{CA}}_{\mathrm{jk}}\right)+r_{1\mathrm{jk}}.$

$\pi_{2\mathrm{jk}}=\beta_{20k}+\beta_{21k}*\left({\mathrm{IV}}_{\mathrm{jk}}\right)+\beta_{22k}*\left({\mathrm{CA}}_{\mathrm{jk}}\right)+\beta_{23k}*\left(\mathrm{IV}*{\mathrm{CA}}_{\mathrm{jk}}\right)+r_{2\mathrm{jk}},$

where *β*_00*k*_, *β*_10*k*_, and *β*_20k_ are intercepts for equations predicting level 1 intercept, linear slope, and quadratic slope, respectively; *β*_01*k*_, *β*_11*k*_, and *β*_21*k*_ are slopes for the effect of initial value (IV) on level 1 intercept, linear slope, and quadratic slope, respectively; *β*_02*k*_, *β*_12*k*_, and *β*_22*k*_ are slopes for the effect of the shock manipulation on level 1 intercept, linear slope, and quadratic slope, respectively; *β*_03*k*_, *β*_13*k*_, and *β*_23*k*_ are slopes for the interaction effect of initial value and shock on level 1 intercept, linear slope, and quadratic slope, respectively; and *r*_0*jk*_, *r*_1*jk*_, and *r*_2*jk*_ are level 2 residual terms.

Level 3 (Individual)

$\beta_{00k}=\gamma_{000}+\gamma_{001}*\left({\mathrm{TA}}_{k}\right)+u_{00k}.$

$\beta_{01k}=\gamma_{010}+\gamma_{011}*\left({\mathrm{TA}}_{k}\right)+u_{01k}.$

$\beta_{02k}=\gamma_{020}+u_{02k}.$

$\beta_{03k}=\gamma_{030}+u_{03k}.$

$\beta_{10k}=\gamma_{100}+\gamma_{101}*\left({\mathrm{TA}}_{k}\right)+u_{10k}.$

$\beta_{11k}=\gamma_{110}+\gamma_{111}*\left({\mathrm{TA}}_{k}\right)+u_{11k}.$

$\beta_{12k}=\gamma_{120}+u_{12k}.$

$\beta_{13k}=\gamma_{130}+u_{13k}.$

$\beta_{20k}=\gamma_{200}+\gamma_{201}*\left({\mathrm{TA}}_{k}\right)+u_{20k}.$

$\beta_{21k}=\gamma_{210}+\gamma_{211}*\left({\mathrm{TA}}_{k}\right)+u_{21k}.$

$\beta_{22k}=\gamma_{220}+u_{22k}.$

$\beta_{23k}=\gamma_{230}+u_{23k},$

where *γ*_000_, *γ*_100_, and *γ*_200_ are level 3 intercepts for equations predicting level 2 intercepts; *γ*_010_, *γ*_020_, *γ*_030_, *γ*_110_, *γ*_120_, *γ*_130_, *γ*_210_, *γ*_220_, and *γ*_230_ are intercepts for equations predicting level 2 slopes; *γ*_001_, *γ*_101_, and *γ*_201_ are slopes for the effect of trait anxiety (TA) on level 2 intercepts; *γ*_011_, *γ*_111_, and *γ*_211_ are slopes for the effect of TA on level 2 slopes; and *u*_00*k*_ through *u*_23*k*_ are residual terms for their respective equations. Of note, the model did not converge when CA was specified as a random effect, so the random effects CA and of interaction terms TA*CA were not included in the final model.
