# Supplementary material for: Anxiety and initial value dependence in startle habituation
Source: Psychophysiology. 2022 Apr 12;59(10):e14071. doi: 10.1111/psyp.14071 (PMC9539862; doi:10.1111/psyp.14071)
Supplement: Supplementary file 1 — TABLE S1 Coding of the time variable for level 1 slopes [file PSYP-59-e14071-s001.docx]

| Suppl. 1  *Coding of the time variable for level 1 slopes* | |
| --- | --- |
| $\left[ \begin{matrix} \begin{matrix} \begin{matrix} 1 & -9.5 & 90.25 \\ 1 & -8.5 & 72.25 \\ 1 & -7.5 & 56.25 \\ 1 & -6.5 & 42.25 \\ 1 & -5.5 & 30.25 \\ 1 & -4.5 & 20.25 \end{matrix} \\ \begin{matrix} 1 & -3.5 & 12.25 \\ 1 & -2.5 & 6.25 \\ 1 & -1.5 & 2.25 \\ 1 & -.5 & 0.25 \\ 1 & .5 & 0.25 \\ 1 & 1.5 & 2.25 \\ 1 & 2.5 & 6.25 \\ 1 & 3.5 & 12.25 \\ 1 & 4.5 & 20.25 \\ 1 & 5.5 & 30.25 \\ 1 & 6.5 & 42.25 \\ 1 & 7.5 & 56.25 \\ 1 & 8.5 & 72.25 \\ 1 & 9.5 & 90.25 \end{matrix} \end{matrix} \end{matrix} \right]$ | From left to right, columns in the matrix represent coding of the time variable for the level 1 model’s calculation of intercept, linear effects, and quadratic effects, respectively. |
